# Containment strategies for the 2019 Novel Coronavirus: flatten the curve or crush it?

**DOI:** 10.1007/s10654-020-00656-x

**Published:** 2020-06-30

**Authors:** Gerry Killeen

**Affiliations:** AXA Research Chair in Applied Pathogen Ecology, School of Biological, Earth and Environmental Sciences, and Environmental Research Institute, University College Cork, Cork, Ireland

Coverage in a national newspaper here in Ireland prompted me to read through the recent epidemiological modelling paper in the *European Journal of Epidemiology* by Dr. Chowdhury and his colleagues in the Global Dynamic Interventions Strategies for COVID-19 Collaborative Group [1]. The modelling approaches taken look solid and reasonable to me, and indeed several of the numerical predictions match our own projections [2]. Worryingly, these similarities including a mean fatality rate of about 1% of the overall population, even in high income countries, in the event of a full-blown, uncontained epidemic. In the Republic of Ireland, that would mean over 40,000 direct COVID-related deaths and obviously many more through indirect effects on health, well-being, social cohesion and economic resilience.

Unfortunately, and consistent with the way their work was represented in one of our national newspapers, the authors only emphasize one side of their own results in the abstract of their paper. There are two issues the authors fail to mention in their own summary: (1) at the end of the 18 month period they presented (Figure 1), epidemics suppressed sufficiently for ICUs to consistently cope with are still going strong, requiring just as much effort to keep contained and continuing to cause illness, death and socioeconomic disruption, (2) when they simulated *crush the curve* [3] approaches to eliminating the virus with sustained and uninterrupted restrictions, their timelines to that exit point are about 3 months (Figure 2), very similar to our own predictions [2, 4].

As explained by Dr. Chowdhury and his colleagues *dynamic interventions* entail repeatedly imposing, lifting and re-imposing restrictions until the epidemic hopefully burns itself out through *herd immunity*, perhaps after 4 years [5], so the graphs presented in figure 1 of their paper [1] probably represents less than half the longer-term picture. Furthermore, assuming the epidemic will indeed burn itself out may prove a dangerous gamble. Instead, it’s likely that COVID-19 establishes itself as a permanently endemic pathogen with volatile and unpredictable dynamics that lead to sporadic epidemics every few years [5]. In the meantime, national strategies intended to *flatten the curve* may well result in a wild roller coaster ride of sequential epidemic surges and re-imposed restrictions that need to be considered before embarking on such a long-term trajectory. On the economic front, such deliberately incomplete suppression of the epidemic means extending the damage over years rather than months [5], asking business to spend more time operating under restrictions that push them into the red than they spend operating anything close to normally and in the black.

So why not just put our foot on the accelerator to terminate the epidemic in time for the coming winter [2–4]? The second set of graphs in figure 2 of Chowdhury et al. [1] almost exactly matches our own [2, 4] and confirms that elimination timelines of 2–4 months are feasible. In the authors’ own words: “…in 3 months, most of the countries would not have any new cases to report”.

Interestingly, the simulations of Chowdhury et al. that achieved such *suppression* assumed a reproductive number of 0.5 [1], almost identical to those documented in Ireland 2 weeks ago, just before we began to relax our restrictions [6]. Taking Ireland as an example, our current rate of epidemic contraction closely matches those predicted by the authors to approach a definitive exit from lockdown within 3 months, an opportunity we may be foolhardy to decline. Faster progress towards elimination would obviously be better and these timelines could be shortened if we were to push ahead with even more stringent and effective restrictions [4]. However, such a bold choice would require a dramatic rethink of our national strategy, broad support from the public at large, and cooperation with our trading partners across Europe and the rest of the world [2]. Open and balanced discourse about the expected long-term consequences of national COVID-19 containment strategies intended to merely *flatten the curve* of epidemics, rather than crush them, is now long overdue in many countries, particularly across western Europe. To avert the worst consequences of further epidemic waves, that discourse now needs progress urgently towards societal consensus within days and weeks rather than months or years.
